# Hair growth promotion by Necrostatin-1s

**DOI:** 10.1038/s41598-020-74796-1

**Published:** 2020-10-19

**Authors:** Mei Zheng, Nahyun Choi, YaeJi Jang, Da Eun Kwak, YoungSoo Kim, Won-Serk Kim, Sang Ho Oh, Jong-Hyuk Sung

**Affiliations:** 1 STEMORE Co. Ltd, Incheon, South Korea; 2 College of Pharmacy, Institute of Pharmaceutical Sciences, Yonsei University, 85 Songdogwahakro, Yeonsu-gu, Incheon, 21983 South Korea; 3 Department of Dermatology, Kangbuk Samsung Hospital, Sungkyunkwan University School of Medicine, Seoul, 03181 South Korea; 4 Department of Dermatology, Severance Hospital and Cutaneous Biology Research Institute, Yonsei University College of Medicine, Seoul, 03722 South Korea

**Keywords:** Cell biology, Molecular biology, Stem cells

## Abstract

Necrostatins (Necs) have been developed as a receptor-interacting protein kinase 1 (RIPK1) inhibitor, thus inhibiting necroptosis. In this current study, we have investigated the possible involvement of necroptosis in the hair cycle regulation and further examined its underlying molecular mechanisms. Diverse RIPK1/3 inhibitors and siRNA were tested in the human outer-root sheath (ORS) cells and animal models. The expression and hair cycle-dependent expression of RIPK 1, respectively, were investigated in the hair follicles (HF) of human, pig, and the mouse. Resulting from the experiment, Nec-1s was most effective in the hair growth promotion among several inhibitors. Nec-1s induced the ORS cell proliferation and migration, and increased the HF length in mouse and pig organ cultures. In addition, it accelerated the telogen-to-anagen transition and elongated the anagen period in the mouse model. Both apoptosis and necroptosis were detected in hair cycle. RIPK1 and RIPK3 were highly expressed in ORS cells during the hair regression period. Nec-1s upregulated the mRNA expression of Wnt3a and Wnt5b, and the activity of β-catenin. Collectively, Nec-1s promotes hair growth through inhibiting necroptosis and activating the Wnt/β-catenin pathway. Necroptosis is involved in hair cycle regression, and Nec-1s is a promising target for hair-loss treatment.

## Introduction

Necrostatin-1 (Nec-1) was first developed as a receptor-interacting protein kinase 1 (RIPK1) inhibitor and has been used to treat other acute central nervous system disorders that feature necroptosis as a mode of cell death^1^. *Takahashi, N. *et al*.,* developed and studied three necrostatin analogs, namely Nec-1, the active inhibitor of RIPK1; Nec-1 inactive (Nec-1i), its inactive variant; and Nec-1 stable (Nec-1s), its more stable variant^2^. Nec-1s has shown superior among other necrostatin analogs in terms of specificity and toxicity. Furthermore, several necrostatin analogs (e.g., sibiriline, GSK'963) have been developed and have contributed to inhibit RIPK1-dependent necroptosis in numerous diseases involving cell death^3,4^. In a preliminary study, we examined the effect of Nec-1s in APP/PS1 mice and found that it improves the treatment of Alzheimer’s disease^5,6^. During that study, we have also found that intraperitoneal Nec-1s injection (100 mg/Kg) promoted hair growth in these aging mice (Fig. S1).

Cell death happens when a biological cell ceases to carry out its functions. Apoptosis and autophagy are forms of programed cell death, whereas necrosis is a non-physiological process that results from an infection or an injury. Though recently, a form of programed necrosis (necroptosis) has been recognized as an alternative form of programed cell death and is associated with death receptors, such as the tumor necrosis factor receptor 1 (TNFR1), followed by the RIPK1 and RIPK3 signaling pathways^7^. The effect of apoptosis on the hair cycle progression has been well-reported. Apoptosis of the outer-root sheath (ORS) plays a key role in the HF regression (catagen phase) regulation^8,9^. For example, apoptotic cells not only appeared in the regressing proximal follicle epithelium but also seen in the central inner root sheath, in the bulge/isthmus region, and in the secondary hair germ, but never in the dermal papillae. Along with catagen stimulatory molecule secretion, ORS cells express apoptotic death receptors, such as Fas, TNFR type 1 receptor (p55), and TGFβ-1/2 receptors^9–12^. However, unlike apoptosis, the involvement of necroptosis or RIPK expression during the hair cycle progression is not well-known. Recently, Jang et al. examined whether or not necroptosis is associated with the pathogenesis of alopecia areata (AA), however, mRNA and protein expressions of RIPK1 and RIPK3 were not upregulated in the skin lesions of patients with AA. We first investigated the effects of necrostatin analogs and RIPK1 inhibition in hair cycle regulation, and have further studied the underlying molecular mechanism, because of the deficient demonstration of the effect of necroptosis on hair cycle progression. The molecular mechanisms and proliferating effect of Nec-1s were assessed in primary human ORS cells. Hair cycle regulation by Nec-1s was analyzed by hair organ culture and several animal experiments. The expression and hair cycle-dependent expression of RIPK1 were investigated in mouse HF.

## Materials and methods

### Cell culture

Human follicle ORS cells at passage (p) 2–4 were obtained (#2420; Sciencell, Carlsbad, CA) and cultured in EpiLife Medium, with 60 µM calcium (Gibco), 1% EpiLife Defined Growth Supplement (EDGS), and 1% Penicillin–Streptomycin (Thermo Fisher Scientific, Waltham, MA, U.S.A.). The cells were maintained in a humidified incubator at 37 °C under 5% CO_2_ and balanced N_2_.

### Proliferation assay

Human ORS cells (passage 3) were plated overnight in triplicate 48-well plates at a density of 6000–7000 cells per well. After 24 h, the culture medium was replaced with a fresh serum-free basal medium containing 1% anti-antibiotics. The cells were incubated in either a vehicle or RIPK inhibitors for 48 h. After incubation, the medium was replaced by CCK-8 solution (Dojindo Molecular Technologies, Inc., Rockville, MD, U.S.A.) followed by an incubation for 2 h. The absorbance was measured at 450 nm using a microplate reader (Tecan, AG, Switzerland).

### Scratch migration assay

5 × 10^5^ cells of ORS (passage 3) were seeded in 35-mm dishes with growth medium. The following day, the growth medium was replaced with basal medium. Confluent cells were wounded using a sterile 1 mL pipette tip and treated with vehicle or Nec-1s. Cell migration was determined via microscopic examination 48 h after wounding. For the evaluation of cell migration, five randomly selected points along each wound were marked and measured the horizontal distances of migrating cells from the wound edge.

### Real-time quantitative reverse-transcription polymerase chain reaction assay

The total cellular RNA was extracted using Invitrogen TRIzol Reagent (Thermo Fisher Scientific), followed by a reverse-transcription using a cDNA synthesis kit (Nanohelix, Daejeon, Korea.). Real-time quantitative reverse-transcription polymerase chain reaction (qRT-PCR) was performed using the StepOne Real-Time PCR System (Applied Biosystems/Thermo Fisher Scientific).

### Human WNT signaling pathway polymerase chain reaction array

The Wnt signaling pathway related genes expression profile of ORS was analyzed using the Human Transduction PathwayFinder RT^2^ Profiler PCR Array (PAHS-043ZA, QIAGEN, Hilden, Germany). Fold changes in expression were calculated using the ΔCt value. Data analysis was based on the ^ΔΔ^CT method with normalization of the raw data to housekeeping genes. The red lines indicate ± 1.5 folds change in gene expression threshold. X axis: genes' expression treated by vehicle treatment, y axis: genes' expression by 100 μM of Nec-1s treatment. Compared with control, Nec-1s upregulated the mRNA expression levels of Wnt 3a, Wnt5b, Wnt6, CCND1, PPARD and downregulated the mRNA expression levels of CXXC4, SFRP1, and DKK1.

### Western blotting

For the preparation of whole-cell extracts, adherent ORS cells (passage 3–4) were washed by PBS, removed by scraping, and lysed in protein extraction solution (INTRON, Seoul, Korea). Total protein and nuclear fractions were separated by sodium dodecyl sulfate–polyacrylamide gel electrophoresis (SDS-PAGE) using 10% gels and transferred to PVDF membranes (Millipore, Bedford, MA). Membranes were blocked with 5% fat-free dried milk in TBS-T (0.1% Tween 20 in Tris-buffered saline) for 1 h at room temperature and then incubated overnight with primary antibody at 4℃. The following day, the membranes were washed thrice with TBS-T and incubated with HRP-conjugated secondary antibody for 1 h at room temperature. The membrane was then reacted with enhanced chemiluminescence solution (Millipore) and photographed. The quantification protein bands was done using Image J. Primary antibodies are presented in Supplementary Table S1.

### Skin biopsies

For depilation experiments, 7-week-old male mice were anesthetized with isoflurane, and hairs in a 2.5 × 4 cm^2^ area of mid-dorsal skin were manually plucked with wax strips to induce synchronized hair cycling. HFs of mice entering telogen were confirmed to change the color of the skin from pink to white. Dorsal skin biopsies were taken from euthanized mice by CO_2_ inhalation. Hairs on the back of mice were carefully shaved using an electric clipper before harvesting skin biopsies. Collected skin tissues were then processed for paraffin sectioning.

### Anagen induction

Mice were maintained and anesthetized according to a protocol approved by the Kim et al.^13^. Pharmacopoeia and the Institutional Animal Care and Use Committee of Yonsei University (IACUC120002). The dorsal area (2.5 cm * 4 cm) of 7-week-old C_3_H/HeN male mice in the telogen stage of the hair cycle were shaved using a clipper and electric shaver, with special care to avoid damaging the bare skin. Topical treatment of control or 0.0003 ~ 0.1% of Nec-1s was done for 2 weeks. Any darkening of the skin (indicative of hair cycle induction) was carefully monitored via photography. After 15 days, dorsal hair was shaved and weighted. The number of anagen hair shafts was counted after haematoxylin and eosin (H & E) staining.

### Anagen elongation

The anagen phase was induced by depilation on the dorsal skin of 7-week-old male mice. All depilated mouse HFs was synchronized in the telogen phase. After 12 days, the animals were topically treated with control, 0.1%, and 0.3% Nec-1s for 5 consecutive days. The dorsal skin of mice was observed, and photographic images were captured 17 days after depilation. The number of anagen and catagen hair shafts was observed by haematoxylin and eosin (H & E) staining. Mice were maintained according to the method previously described by Puas et al.^14^. See Experimental scheme (Fig. 2E).

### Hair organ culture

The hair growth activity of mouse vibrissae and pig back hair was observed during organ culture. The HFs were isolated and cultured according to the method previously described by Jindo and Tsuboi^15^. Normal anagen vibrissal HFs were obtained using a scalpel and tweezers from the upper lip region. Isolated vibrissal HFs of mice were placed in defined medium (Williams E medium supplemented with 2 mM L-glutamine, 10 µg/mL insulin, 10 ng/mL hydrocortisone, 100 U/mL penicillin, and 100 µg/mL streptomycin, without serum) with vehicle or RIPK inhibitors. Individual HFs were photographed 72 h after the start of incubation (Edmund Optics Ltd, UK). Changes in hair length were calculated from the photographs and expressed as mean ± SE of 10–12 vibrissal HFs.

### Immunofluorescence analysis of cleaved caspase-3 and in situ fluorescent TUNEL staining

Mouse skin tissue slides were de-paraffin and incubated with antigen unmasking solution supplemented with 20 μg/mL proteinase K for 5 min at RT. The slides were then washed with PBS and incubated with 4% formaldehyde/PBS for 5 min at RT. After washing with PBS, the samples were next incubated with the in situ cell death detection kit reagents (ab66110, Abcam, Cambridge, MA, USA) according to the manufacturer's instructions. The slides were then washed with PBS and incubated with a rabbit polyclonal antibody against cleaved caspase-3 overnight at 4℃ and subsequently incubated with Alexa Fluor 488-conjugated goat anti-rabbit IgG. Nuclei were counterstained using 4′,6-diamidino-2-phenylindole (DAPI; Sigma). The TUNEL-positive cells and the number of TUNEL-positive cells that expressed or did not express cleaved caspase-3 were detected using fluorescence microscopy (Eclipse E600).

### Knockdown of RIPK in vivo

RIPK1 and RIPK3 were silenced in C_3_H mice in vivo by transfecting with 6 μg of RIPK1 vs. negative control siRNA (Bioneer, Daejeon, Korea) using Polyplus in vivo-jetPEI (Polyplus-transfection, Illkirch, France) as a transfection reagent with a transfection efficiency of ~ 70% as previously described^16,17^.

*See* Experimental scheme (Fig. 5B).

### β-catenin promoter activity assay in vitro

Human HaCaT cell and 293 T cell which stably expressed β-catenin promoter-Luc, were seeded in 24-well plates at 80% confluence. The cells were treated with indicated concentrations of Nec-1s for 24 h and then collected in 1 × passive lysis buffer (Promega). The luciferase assay as conducted using the Luciferase Reporter Assay System (Promega) followed the manufacturer’s recommendations. Luminescence was measured using a Glomax Discover microplate Reader (Promega).

### Statistical analysis

All data are expressed as the mean ± SD of three independent experiments. Differences between the treatment groups were evaluated using one-way ANOVA or Student’s *t*-test.

## Results

### Nec-1s facilitates hair growth in hair organ culture and hORS cell proliferation

**Figure 1:**
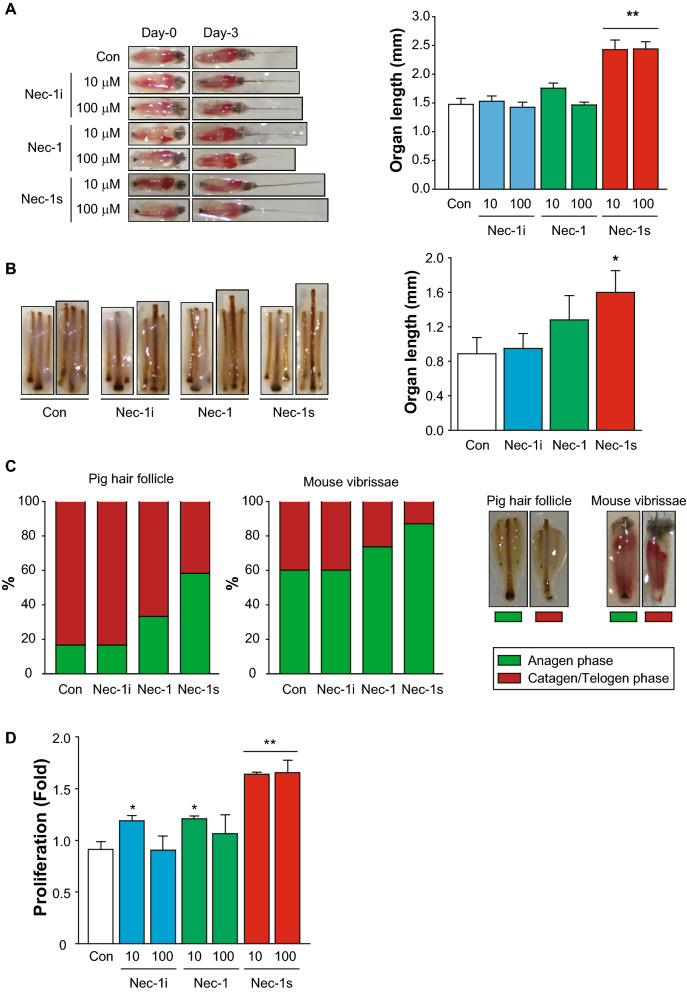
Hair growth-promoting effects of necrostatin derivatives. Among the necrostatin analogs (Necs), Nec-1s treatment significantly promoted mouse vibrissa follicle (**A**) and pig HFs (**B**) growth ex vivo. ** *p* < 0.01; * *p* < 0.05, n = 10–12 mouse vibrissa follicles per group, n = 8 pig HFs per group. (**C**) The number of pig HFs and mouse vibrissae follicles in the anagen and non-anagen phase was counted. Anagen and non-anagen pig HF or mouse vibrissae follicle was observed (C, right panel). Anagen HFs (green box) show a hair matrix (HM) with a large volume and a melanin content which is maximal whereas catagen/telogen HF (red box) have a thinner HM and reduced melanin content. (**D**) The proliferation of outer-root sheath (ORS) cells was measured. White bars: control, blue bars: Nec-1i, green bars: Nec-1, red bars: Nec-1s.

We first evaluated the hair growth-promoting effect of the three Nec analogs in the mouse vibrissa organ culture. Only Nec-1s increased the length of the mouse vibrissae HF at day 3 (Fig. 1A). Also, only Nec-1s increased the hair length in the pig hair organ culture (Fig. 1B). The percentage of pig skin and mouse vibrissae HF in the anagen phase was higher in Nec-1s than other Nec analogs (Fig. 1C). In order to distinguish between anagen (green box) and catagen/telogen (red box), the qualitative morphological criteria were shown on right panel of Fig. 1C. In HF organ culture anagen, HFs showed a hair matrix (HM) with a large volume and a melanin content which is maximal whereas catagen/telogen HF had a thinner HM and reduced melanin content. In addition to that, Nec-1s significantly enhanced the proliferation of ORS cells (Fig. 1D).

### Nec-1s regulates hair cycle

**Figure 2:**
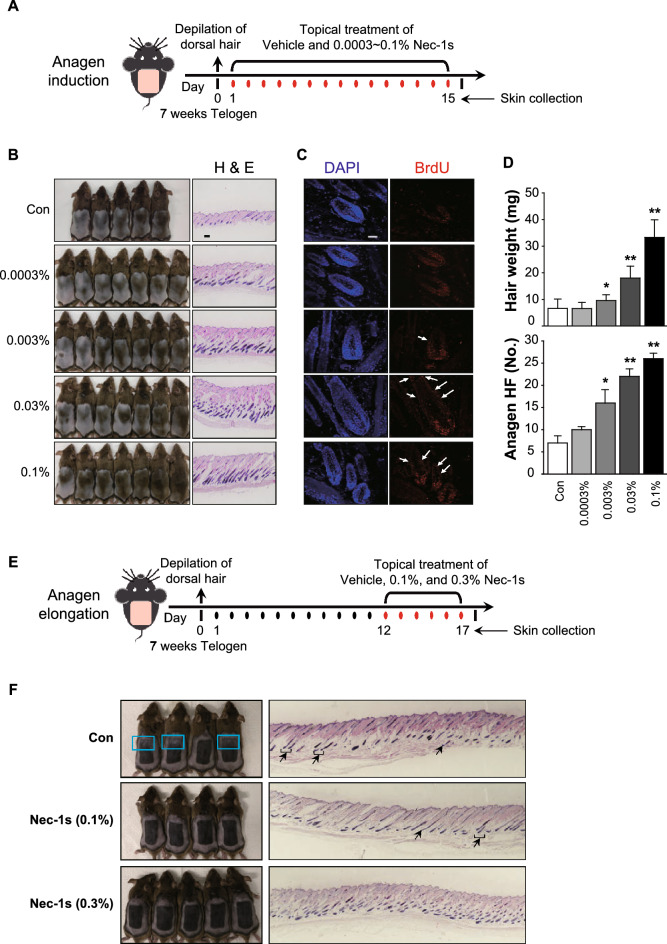
Hair growth-promoting effects of necrostatin-1s. (**A**) The experiment design. The back skin of 7-week-old C_3_H mice was shaved, and 0.0003%–0.1% Nec-1s was topically applied. (**B**) Compared with the negative control, topical application of Nec-1s (0.003%–0.1%) facilitated the telogen-to-anagen transition in C_3_H mice at day 15. Hematoxylin and eosin (H&E)-stained tissues revealed more mature HFs, and the number of proliferating hair matrix cells and ORS cells (BrdU-immunopositive cells) also increased in the skin of Nec-1s-treated mice (**C**,**D**). (**E**) Anagen elongation experiment design. (**F**) Photographs were taken after topical application of Nec-1s for 5 days. More catagen/telogen follicles were found in control mice (as indicated by the arrow in H&E staining). Compared with the control group (blue boxes indicate catagen/telogen phase,), Nec-1s elongated the anagen phase in C_3_H mice. Scale bar = 50 μm. * *p* < 0.05. ** *p* < 0.01.

We used telogen phase 7-week-old male C_3_H mice to investigate the telogen-to-anagen transition (Fig. 2A)^18^. Compared with the negative control, topical application of Nec-1s (0.003%–0.1%) facilitated the telogen-to-anagen transition in C_3_H mice at day 15 (Fig. 2B). Further examination of hematoxylin and eosin (H&E)-stained tissues revealed more mature HFs, as observed in Nec-1s-treated mice. Hair matrix cell and ORS cell proliferation in the HFs (BrdU-immunopositive cells) were also increased in the skin of Nec-1s-treated mice (Fig. 2C). The hair weight and anagen HF number were significantly increased by Nec-1s in a dose-dependent manner (Fig. 2D). We next investigated whether Nec-1s can prolong the anagen phase of the hair cycle in C_3_H mice (Fig. 2E). More catagen HFs was found in control-treated mouse, and the anagen phase was prolonged by topical treatment of Nec-1s (0.1% and 0.3%) for 5 days (Fig. 2F).

### RIPK1 and RIPK3 are expressed in outer-root sheath

**Figure 3:**
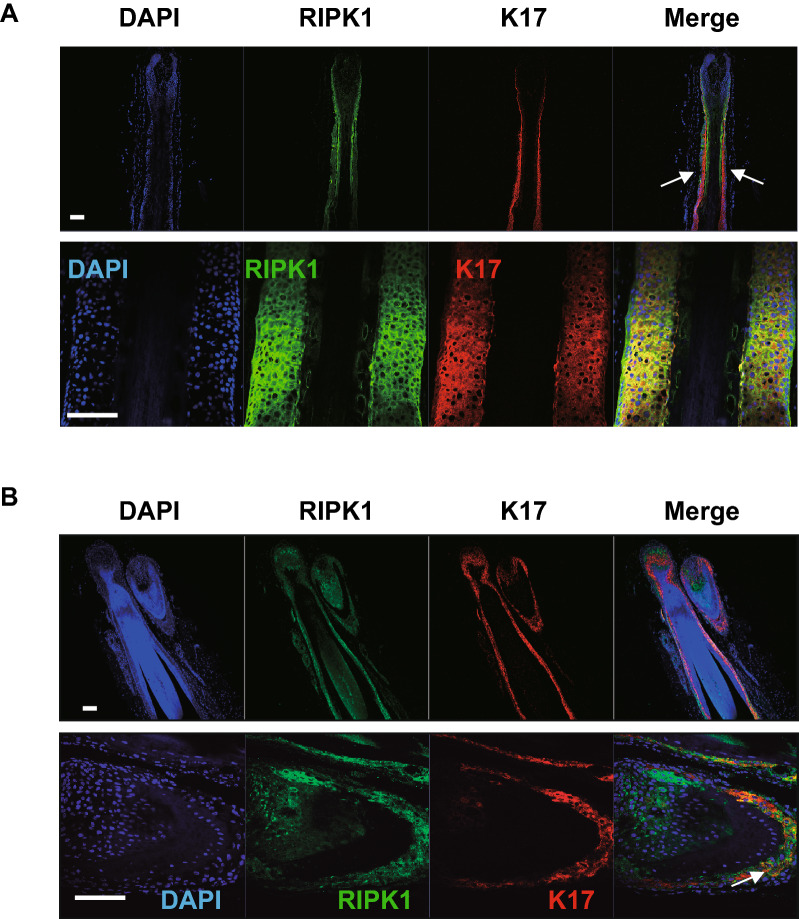
RIPK1 expression in the hair follicle. (**A**) Human HFs were kindly received from Prof. Sang Ho Oh (*Severance Hospital, Yonsei, University College of Medicine*) and (**B**) pig HFs were isolated from 1-week-old pig back skin. Immunostaining showed that RIPK1 (green) localized in the outer-root sheath (ORS) region of HFs and co-localized with keratin 17 (k17, blue, an ORS marker) in the HF. DAPI staining (blue) indicates nuclei. Scale bar = 50 μm.

Nec-1s had been developed to inhibit RIPK1-dependent necroptosis ^7,19–22^. Therefore, we examined where RIPKs are expressed in the HF and analyzed RIPK1 and RIPK3 proteins by immunofluorescence in human and pig HFs. Both RIPK1 and RIPK3 were highly expressed in the ORS region of human scalp HFs and ORS region of pig HFs which were indicated by co-staining with keratin 17 (k17, an ORS marker) in pig HF (Figs. 3A,B and S2A,B). These results suggested that Nec-1 s may target RIPK1 and RIPK3 in the ORS region.

### Necroptosis occurred during mouse hair cycle

**Figure 4:**
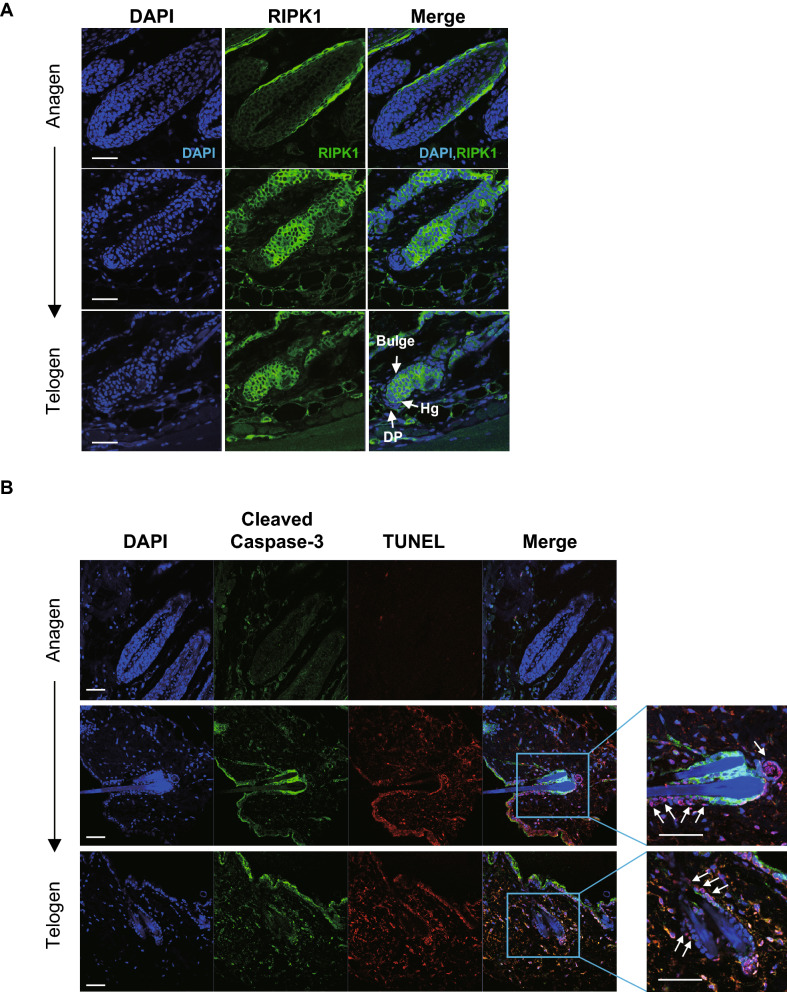
Necroptosis occurred during mouse hair cycle. The paraffin sections were isolated from 4-, 5-, and 7-week-old C_3_H mice. (**A**) Immunofluorescent detection of RIPK1 (green) in mouse dorsal skin. RIPK1 was expressed in the ORS region during the anagen phase and increased during the catagen/telogen phase at the hair germ and bulge area. DAPI staining (blue) indicates nuclei. DP, dermal papillae; HG, hair germ. Scale bar = 50 μm. (**B**) TUNEL and cleaved caspase-3 dual immunofluorescent labelling in the skin tissue. TUNEL (Red) and cleaved caspase-3 (Green) staining were performed. Both TUNEL‐ and cleaved caspase‐3‐positive cells were apoptotic, while TUNEL‐positive but cleaved caspase‐3‐negative cells were necroptic. The TUNEL^+^/c-cas3^-^ cells were found in ORS of the catagen/telogen HFs (as indicated by the arrows). Scale bar = 100 μm.

In our study, RIPK1 was expressed in ORS region of HFs at anagen stage of human, pig and mice (Fig. 3 and 4A), and was also expressed in hair germ cells and bulge region cells, which are the cells that will later differentiate ORS, at catagen/telogen stage, not in the DP region (Fig. 4A). In addition, the expression level of RIPK1 in ORS region was highest at catagen (Fig. 4A) suggesting that RIPK1-related cell death may occur during normal hair cycle, especially at catagen stage. To investigate which cell death occurred during hair cycling, we performed double labelling with TUNEL and cleaved caspase-3 (c-cas3) during hair cycling. TUNEL-positive and c-cas3-positive (TUNEL^+^/c-cas3^+^) cells were defined as apoptotic cells, and TUNEL-positive but c-cas3-negative (TUNEL^+^/c-cas3^-^) cells were known as necroptotic cells^23,24^. According to our result on Fig. 4B, the number of TUNEL^+^/c-cas3^+^ cells (apoptotic cells) were highest at catagen and the number of apoptotic cells was decreased at telogen stage, suggesting that apoptosis was occurred most often during catagen and slowly decreased at telogen stage. Although necroptosis was occurred at a smaller rate compared to apoptosis at catagen/telegen, TUNEL^+^/c-cas3^-^ cells (necroptotic cell) existed in ORS cells of HFs and highest at catagen stage (Fig. 4B, arrows). These results suggested that both apoptosis and necroptosis occurred in mainly ORS cells at catagen stage during normal hair cycling, and necroptosis still occurred at telogen stage. RIPK3 was difficultly detected in the HFs in the anagen phase, and RIPK3 was weakly expressed in the hair germ and bulge region at catagen/telogen phase (Fig. S2C). Therefore, we can conclude that necroptosis occurred together with apoptosis during the normal mouse hair cycle, and RIPK1/RIPK3 might be involved in the regulation of mouse hair cycle.

### Inhibition of RIPKs promotes hair growth

**Figure 5:**
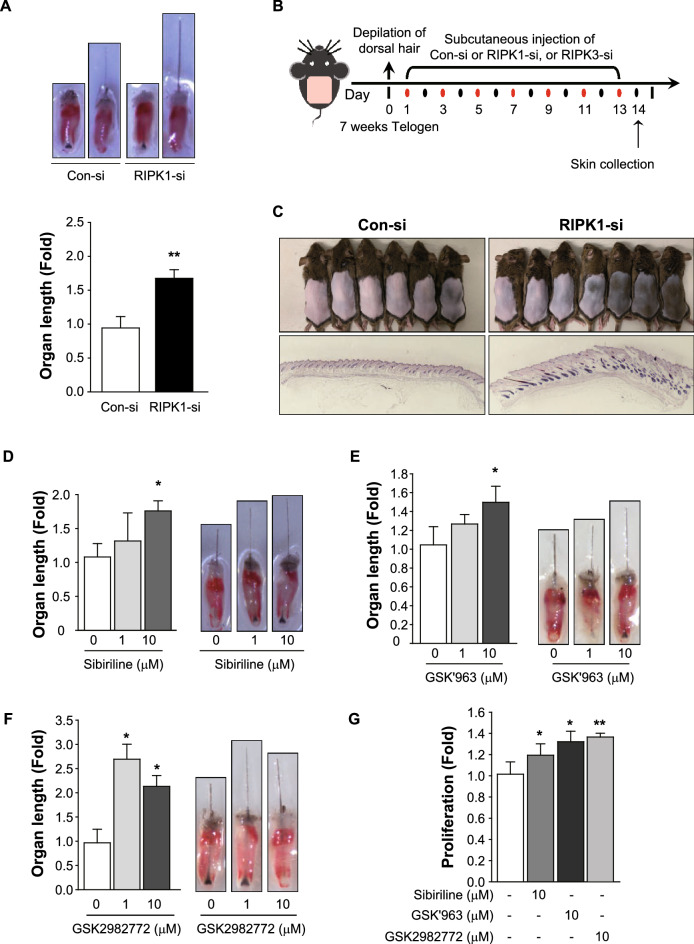
Hair growth-promoting effects of RIPK inhibition. (**A**) Mouse vibrissa follicles were isolated and cultured with 1 μg of RIPK1 siRNA treatment for 3 days. RIPK1 knockdown increased hair fiber length in the organ culture. (**B**) The experiment design. After depilation of the dorsal skin, 6 μg of siRNA was injected for every 2 days. Injection of siRNA for RIPK1 accelerated the telogen-to-anagen transition in C_3_H mice (**C**). (**D–F**) Hair growth-promoting effect of several RIPK1 inhibitors. Sibiriline (**D**), GSK'963 (**E**), or GSK2982772 (**F**) treatment significantly promoted mouse vibrissa follicle growth ex vivo. They also increased hORS cell proliferation (**G**). * *p* < *0.05*, ** *p* < *0.01*. n = 10–12 mouse vibrissa follicles per group.

We further studied whether RIPK1 or RIPK3 inhibition would enhance the hair growth. In the vibrissa organ culture, both treatments of RIPK1 siRNA and RIPK3 siRNA promoted mouse vibrissa length for 1.5–2 folds (Figs. 5A and S2D). Furthermore, siRNA injection for RIPK1 and RIPK3 accelerated telogen-to-anagen transition in C_3_H mice (Figs. 5B,C, S2E). In addition, RIPK1 pharmacological inhibition was tested. Sibiriline, GSK᾽963, and GSK2982772 treatment significantly elongated hair length in the mouse vibrissa organ culture (Fig. 5D–F) and increased human ORS cell proliferation (Fig. 5G). These results indicated that RIPK1 inhibitors promote hair growth by inhibiting necroptosis.

### Wnt/β-catenin signaling is activated by Nec-1s in hORS cells

**Figure 6:**
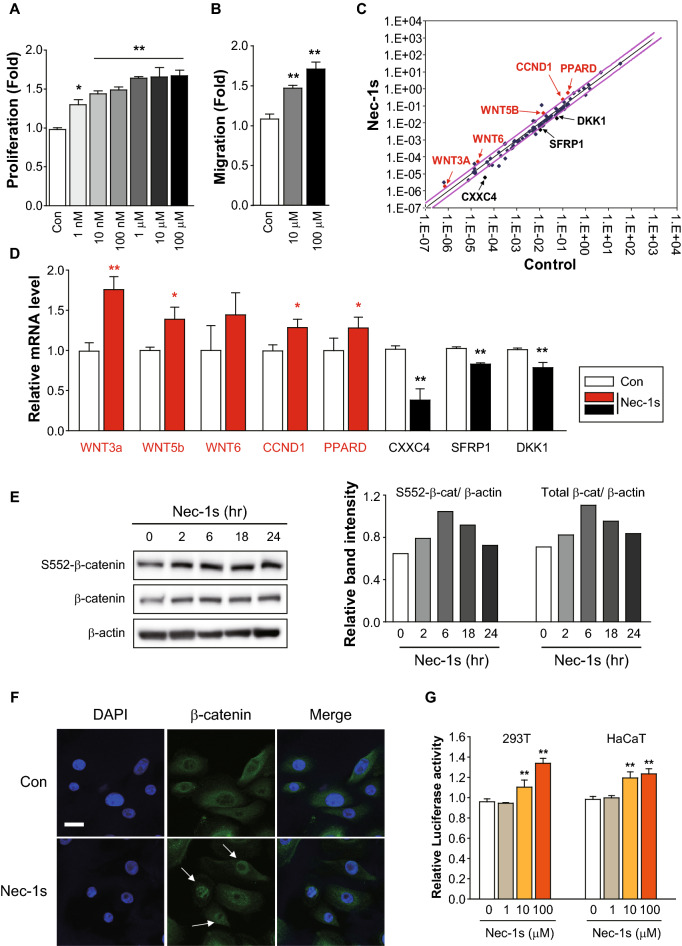
Involvement of Wnt/β-catenin signaling pathway. (**A,B**) Nec-1s treatment increased hORS cell proliferation (**A**) and migration (**B**) in a dose-dependent manner. (**C**) Wnt signaling pathway was observed using a RT^2^ profiler PCR assay. Among the 84 Wnt-related genes, five genes were increased (Wnt3a, Wnt5b, Wnt6, CCND1, and PPARD) and three (CXXC4, SFRP1, and DKK1) were decreased. (**D**) qRT-PCR was used to confirm upregulation and downregulation of mRNA expression. (**E**) Nec-1s (100 μM) increased the total and active β-catenin (ser552) protein expression in ORS cells, the blot density was quantified by Image J. (**F**) β-catenin immunostaining was carried out in hORS cells. β-catenin (green) translocation from the cytosol to the nucleus was detected in Nec-1s-treated cells (arrowhead). Scale bar = 50 μm. (G) Nec-1s enhance b-catenin promoter activity. A luciferase assay was performed by Human HaCaT and HEK293T cell, which stably expressed β-catenin-Luc. Nec-1s treatment resulted in a 40% increase in β-catenin promoter activity. * *p* < *0.05*, ** *p* < *0.01*. Full-length original blots are shown in Fig. S5.

ORS cells were treated with Nec-1s (1 nM–100 μM) for 48 h, and both ORS proliferation and migration were enhanced by Nec-1s in a dose-dependent manner (Fig. 6A,B). Therefore, we explored the underlying mechanism of hair growth promotion by Nec-1s in hORS cells. In a previous study, Nec-1s led to an increased Wnt3a^25^. We hypothesized the Wnt/β-catenin pathway involvement in hair growth. We measured the upregulated mRNA levels for the Wnt signaling pathway using PCR array. Five genes were increased (Wnt3a, Wnt5b, Wnt6, CCND1, and PPARD) and three were significantly decreased (CXXC4, SFRP1, and DKK1) (fold change > 1.5) among the 84 Wnt-related genes in human ORS cells (Fig. 6c), and the mRNA expression levels were further verified by qRT-PCR (Fig. 6d). Furthermore, Nec-1s increased active β-catenin (ser552) expression, which protects its degradation from GSK3β and promotes its translocation into the nucleus (Fig. 6E). Therefore, Nec-1s treatment increased β-catenin accumulation in nucleus of ORS cells (Fig. 6F). The direct effect of the treatment with Nec-1s on β-catenin activity was confirmed by a β-catenin promoter-Luciferase activity assay in human HaCaT and 293 T cells (Fig. 6G). Taken together, these results indicate that Nec-1s activates ORS cells via activating the Wnt/β-catenin signaling pathway.

## Discussion

We first investigated whether or not Nec analogs and RIPK1 inhibitors promote hair growth and further examined the underlying molecular mechanisms in the present study. Nec-1s was the most effective hair growth promoter among the Nec derivatives. Nec-1s induced ORS cell proliferation and increased the HF length in the mouse and pig organ cultures. In addition, it accelerated the telogen-to-anagen transition and anagen elongation in C_3_H mice.

We measured the expression of RIPK1 during the anagen to telogen phase in C3H mice, and RIPK1 was highly expressed in ORS cells during the hair regression period (catagen and telogen phases). We observed an increase of apoptosis and necroptosis in ORS cells at catagen stage by TUNEL/cleaved caspase-3 double staining, and still observed necroptotic cells at telogen stage. The transection of RIPK1- or RIPK3-siRNA increased HF length in hair organ cultures, and accelerated telogen-to-anagen transition in the animal model. Nec-1s upregulated Wnt3a and Wnt5b mRNA expression and increased the translocalization of β-catenin into the nucleus by stimulating β-catenin promoter binding activity. Collectively, these results indicated that Nec-1s has hair growth promoting effects, and necroptosis is involved in normal hair cycle regulation.

Jang et al*.* examined whether or not necroptosis is associated with the pathogenesis of AA, and found that RIPK1 and RIPK3 mRNA and protein expressions were not upregulated in the skin lesions of AA patients^26^. The expression of necroptosis-related genes (RIPK1, RIPK3, and MLKL) was also highly expressed in the epidermis of human, whereas RIPK1 expression was high in the HF of BALB/c mice^24^. They also reported that keratinocyte necroptosis inhibition prevented psoriatic inflammation, and both the inhibitors of RIPK1 (Nec-1s) and MLKL (e.g., necrosulfonamide) suppressed necroptosis in HaCaT cells. RIPK1 was highly expressed in the epidermal basal layer, although its expression decreased significantly in the skin lesions of psoriasis vulgaris. We also found that RIPK1 was highly expressed in epidermal layers and in the outer layer of HFs (ORS cells, bulge, and hair germ cells). Therefore, it is reasonable to assume that RIPK1 may regulate hair cycle regression by inducing epidermal cell death in HFs.

RIPK1 mediates apoptosis and necroptosis, and both are important for late embryonic development and the prevention of inflammation in epithelial barriers^27–29^. RIPK1 mediates inflammatory and cell death signaling, and drives RIPK3 and MLKL-mediated necroptosis. For example, Dannappel et al. reported that skin inflammation in RIPK1 knockout mice was dependent on RIPK3-mediated necroptosis and MLKL CRISPR/Cas9-mediated knockouts prevent skin inflammation in RIPK1 knockout mice^28^. Lin et al. also showed that epidermal RIPK1 knockouts induced skin inflammation by inhibiting Z-DNA-binding protein 1 (ZBP1)-mediated activation of RIPK3/MLKL-dependent necroptosis^30^. Duan et al., found that Nec-1s affected programed necrosis, rather than apoptosis, in IMQ-induced psoriasiform dermatitis in mice by detecting TUNEL-stained cells and cleaved caspase-3 staining. Of interest, RIPK3 kinase activity is essential for necroptosis and governs whether a cell activates caspase-8 and dies by apoptosis^31^. We found in the present study that Nec-1s inhibited TNF-α- and LPS-induced RIPK1 and RIPK3 phosphorylation; however, it did not attenuate cleaved caspase-3 formation (Fig. S3). Moreover, our double labelling results with TUNEL and cleaved caspase-3 (c-cas3) during hair cycling revealed that although necroptosis was occurred at a smaller rate compared to apoptosis, necroptotic cells existed in ORS cells of HFs and highest at catagen/telogen stage (Fig. 4B). These results indicated that both apoptosis and necroptosis occurred in mainly ORS cells at catagen stage during normal hair cycling, and necroptosis still occurred at telogen stage. These results correspond to the result that expression level of RIPK1 was highest at catagen stage and decreased at telogen stage (Fig. 4A). These results suggested that apoptosis by RIPK1 may occur mainly at catagen stage, but necroptosis by RIPK1 may occur at both catagen and telogen stages. The reason why RIPK1 still being expressed at telogen in bulge cell and hair germ, is probably because necroptosis was occurring to some extent until telogen stage though further study may be needed. Therefore, it is reasonable to assume that hair cycle regression is partly mediated by RIPK1/3-dependent necroptosis.

There are several evidences that the Wnt/β-catenin signaling pathway plays a key role in hair morphogenesis and hair cycle progression^32^. For example, LEF/TCF DNA-binding proteins act together with activated β-catenin to transactivate downstream target genes to promote hair growth^33^. Wnt signaling inhibition after re-epithelialization completely abrogated the wounding-induced folliculogenesis, whereas Wnt ligand overexpression increased the number of regenerated HFs^34^. Wnt ligand expression is increased within the regenerating epithelium during the anagen phase induction and plays a key role in epidermal stem cell activation^35^. Of note, Nec-1s led to increased neurite number in the retinal ganglion cell via Wnt3a-dependent regulation^25^. Therefore, we hypothesized that Nec-1s promotes hair growth via the Wnt signaling pathway. As expected, Nec-1s upregulated the WNT3A, WNT 5A, WNT6, CCND1, and PPARD, whereas it downregulated CXXC4, SFRP1, and DKK1, and thereby translocated β-catenin into the nucleus. It is reasonable to assume that Nec-1s promotes hair growth via Wnt/β-catenin signaling pathway activation.

Necroptosis has an important role in the programed cell death in embryonic development, tissue homeostasis, immunity, and inflammation^36^. RIPK1-mediated cell death leads to epithelial barrier disruption and/or release of damage-associated cytokines and chemokines, which mediate inflammatory and degenerative diseases^37^. Therefore, many pharmaceutical companies have pursued targeting RIPK1 and, to a lesser extent, RIPK3 for drug development. For example, GSK2982772 entered clinical trials for inflammatory disorder treatment, and blood–brain barrier-permeable DNL747 passed phase I clinical trials for the treatment of Alzheimer’s disease, amyotrophic lateral sclerosis, and multiple sclerosis^38,39^. Novel small-molecule drugs were classified and existed based on their pharmacodynamic type1–3 binding mode^22,37^. Nec analogs are the first RIPK1 inhibitors to be discovered with an ATP-competitive type 3 binding mode, and we further tested three type 3 inhibitors for hair-loss treatment (Figs. 1 and 5). Most RIPK1 inhibitors were effective, although Nec-1s exhibited the strongest hair growth-promoting effect in vitro and in animal experiments. More importantly, the intraperitoneal (100 mg/kg) and topical application (0.03–0.3%) of Nec-1s was effective for hair growth in various animal experiments (Fig. 2, and Fig. S1). Therefore, we can conclude that RIPK inhibitors, such as Nec-1s, can be a new target for hair-loss treatment (Fig. S4).

## Supplementary information

Supplementary Information.
