# Author Correction: Hair growth promotion by Necrostatin-1s

**DOI:** 10.1038/s41598-021-88759-7

**Published:** 2021-04-21

**Authors:** Mei Zheng, Nahyun Choi, YaeJi Jang, Da Eun Kwak, YoungSoo Kim, Won-Serk Kim, Sang Ho Oh, Jong-Hyuk Sung

**Affiliations:** 1 STEMORE Co. Ltd, Incheon, South Korea; 2 College of Pharmacy, Institute of Pharmaceutical Sciences, Yonsei University, 85 Songdogwahakro, Yeonsu-gu, Incheon, 21983 South Korea; 3 Department of Dermatology, Kangbuk Samsung Hospital, Sungkyunkwan University School of Medicine, Seoul, 03181 South Korea; 4 Department of Dermatology, Severance Hospital and Cutaneous Biology Research Institute, Yonsei University College of Medicine, Seoul, 03722 South Korea

Correction to: *Scientific Reports* 10.1038/s41598-020-74796-1, published online 19 October 2020

The original version of this Article contained an error in the title of the paper, where the word “Necrostatin” was incorrectly given as “necrostatin”. This has now been corrected in the PDF and HTML versions of the Article, and in the accompanying Supporting Information file.

Furthermore, there was an error in Affiliation 2, which was incorrectly given as ‘College of Pharmacy, Yonsei Institute of Pharmaceutical Sciences, Yonsei University, 85 Songdogwahakro, Yeonsu-gu, Incheon 21983, South Korea’. The correct affiliation is listed below:

College of Pharmacy, Institute of Pharmaceutical Sciences, Yonsei University, 85 Songdogwahakro, Yeonsu-gu, Incheon 21983, South Korea

