# Supplementary material for: Hair growth promotion by Necrostatin-1s
Source: Sci Rep. 2020 Oct 19;10:17622. doi: 10.1038/s41598-020-74796-1 (PMC7573580; doi:10.1038/s41598-020-74796-1)
Supplement: Supplementary file 1 — Supplementary Information. [file 41598_2020_74796_MOESM1_ESM.pdf]

Hair growth promotion by Necrostatin-1s

Mei Zheng, Nahyun Choi, YaeJi Jang, Da Eun Kwak, YoungSoo Kim, Won-Serk Kim, Sang Ho Oh, Jong-Hyuk Sung

Antibodies	Species	Source	Dilution (IF)	Dilution (WB)	Used to identify
Keratin 17	Mouse	Santa cruz	1:100		Hair follicle
RIPK1	Rabbit	Novus biologicals	1:100	1:1000	Hair follicle
RIPK3	Rabbit	Abcam	1:100	1:1000	Hair follicle
^{S161}RIPK	Rabbit	Invitrogen	1:100	1:1000	Hair follicle & ORS cell
^{S552}β-catenin	Rabbit	Cell signaling		1:1000	ORS cell
β-catenin	Rabbit	Cell signaling	1:100	1:1000	ORS cell
Caspase-3	Rabbit	Cell signaling		1:1000	ORS cell
Cleaved-caspase3	Rabbit	Cell signaling	1:100	1:1000	Hair follicle & ORS cell
β-catenin	Mouse	Santa cruz		1:2000	ORS cell
BrdU	Mouse	Abcam	1:100		Hair follicle

Table S1. Primary antibodies used for immunofluorescence analysis and western blot.

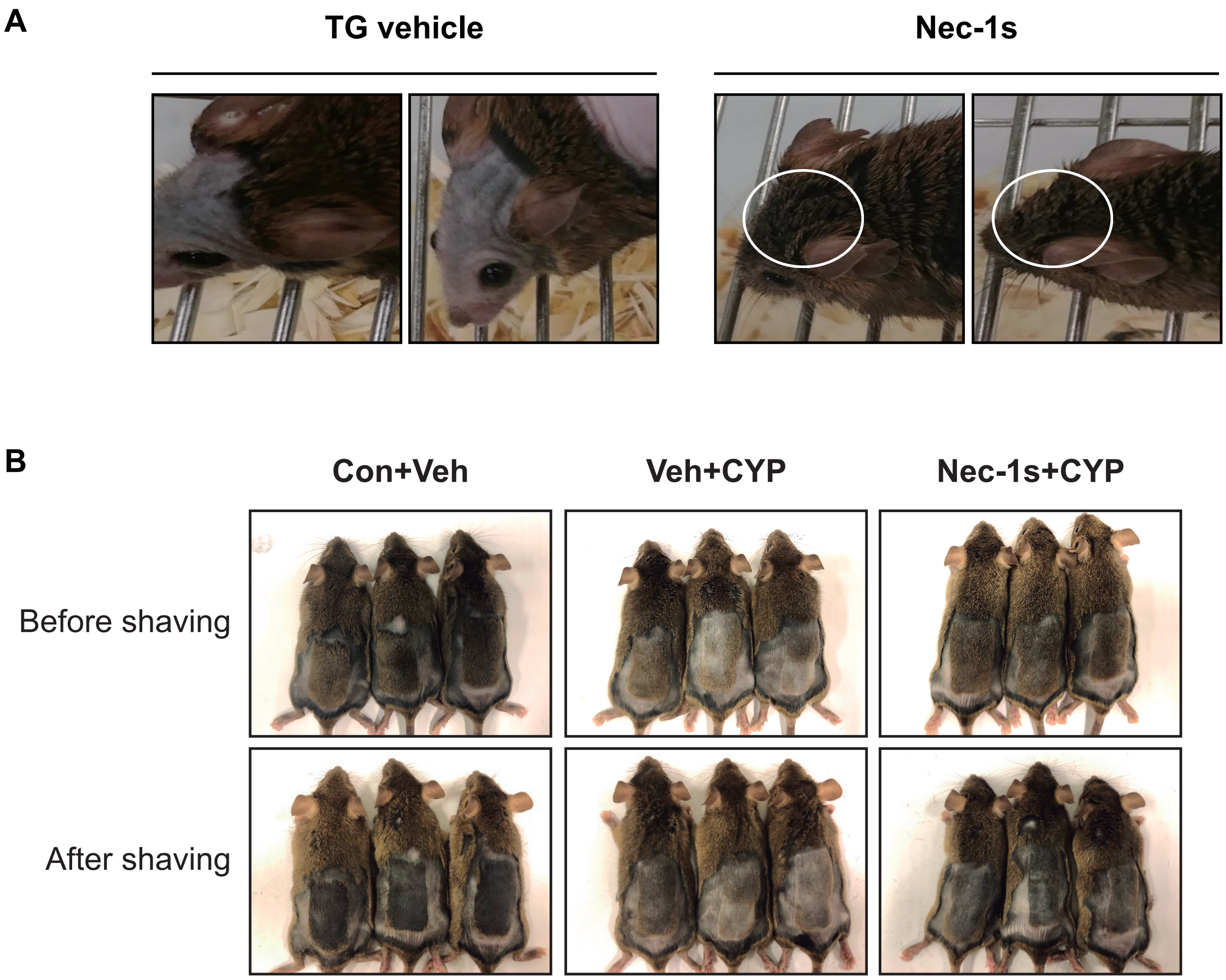

Figure S1. Nec-1s treatment regrow hair in AD and CYP model

(a) Nec-1s (100 mg/kg) or vehicle (2.5% DMSO in PBS) was injected into 8-month-old male APP/PS1 mice via tail vein for 4 weeks (2 times per week). Photographic images were captured after 4 weeks.
(B) 7-week-old mice were anesthetized with isoflurane, and hairs in a 2.5 x 4 cm^2 area of mid-dorsal skin were manually plucked with wax strips to induce synchronized hair cycling. 125 mg/kg of cyclophosphamide (CYP) was subcutaneous injected at day 8 after depilation. 0.1% of Nec-1s was topical treated from day 9 to day 16, photographic images were captured at 17 days after depilation.

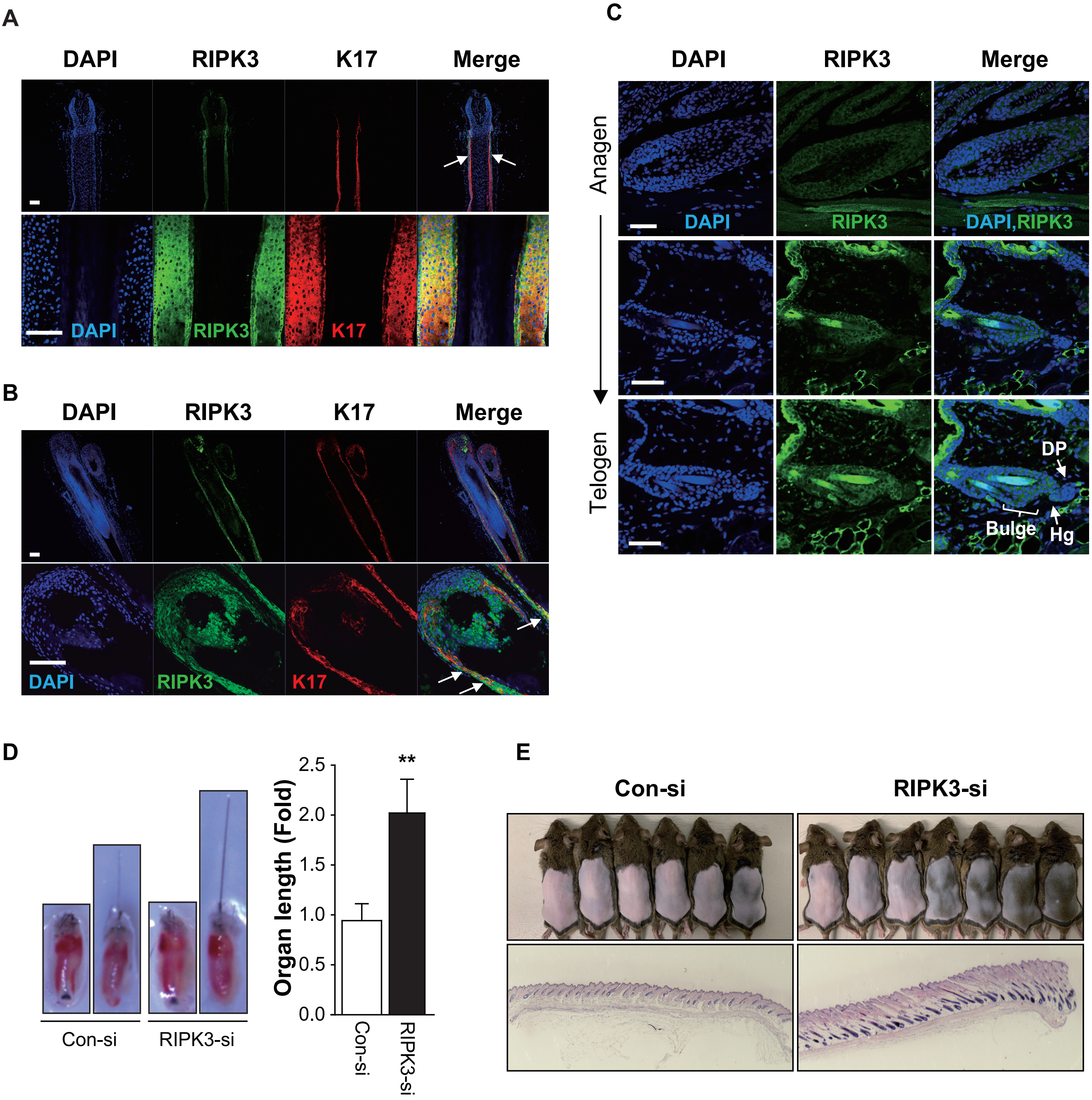

Figure S2. RIPK3 expression in the ORS of human and pig hair follicle; Knockdown of RIPK3 promotes hair growth *in vivo*.

Immunostaining showed that RIPK3 (green) localized in the ORS region of human HF **(a)** and pig HF **(b)** and co-localized with keratin 17 in the HF. **(c)** RIPK3 was difficultly detected in the HFs in the anagen phase, and RIPK3 was weakly expressed in the hair germ and bulge area during the catagen/telogen phase. DAPI staining (blue) indicates nuclei. DP, dermal papilae; HG, hair germ. Scale bar = 50µm. ** *p<0.01*. n=10-12 mouse vibrissa follicle per group.

Supplementary Figure 3

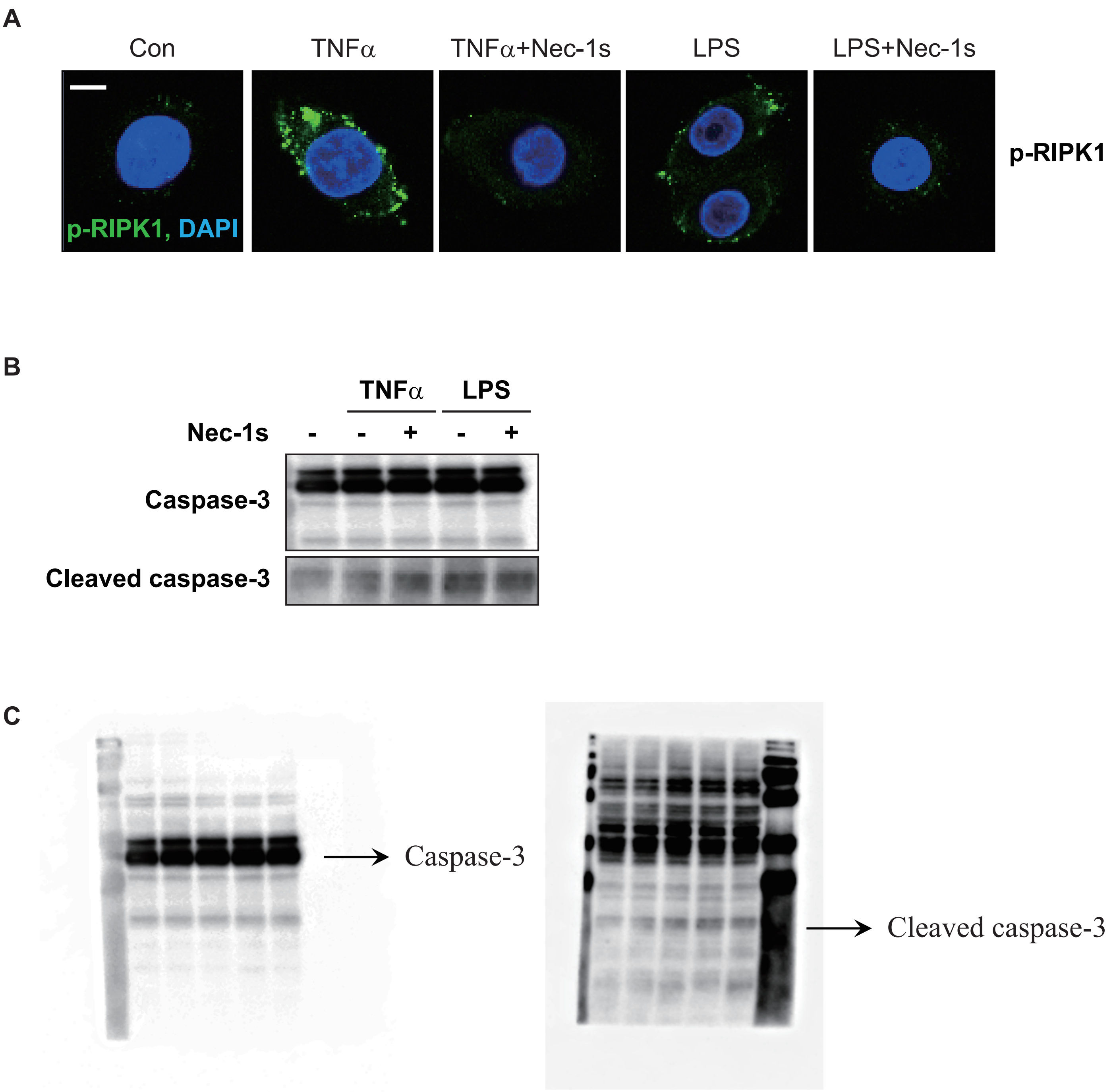

Figure S3. Nec-1s blocks TNFa and LPS-induced phosphorylation of RIPK; Nec-1s blocked necroptosis rather than apoptosis in ORS cells.

(A) ORS cell were incubated under 1 or 10 ng/ml of TNFa or LPS for indicated time. ORS were pre-teated with 100 mM of Nec-1s for 1 hr at 37 degree, followed by TNFa or LPS stimulation. The level of p-RIPK1 was detected by immunostaing. (B) protein level of total caspase-3 and cleaved caspase-3 were analyzed by Western blotting in ORS cells. ORS cell passage=3. Full length orginal blots are shown in (C).

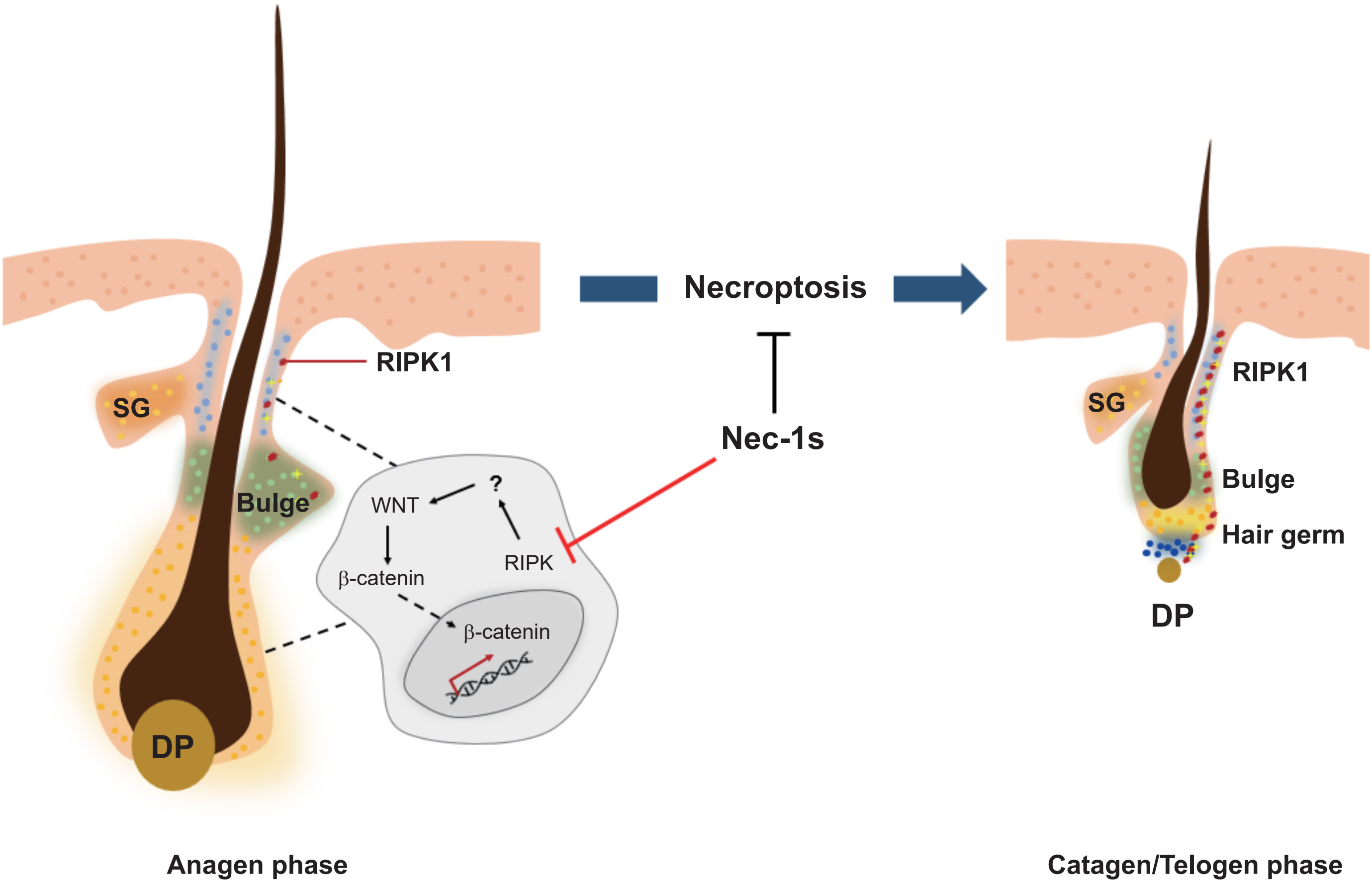

Figure S4. Model for Nec-1s function in the regulation of hair growth

Supplementary Figure 5

Full-length blots related to Figure 6E

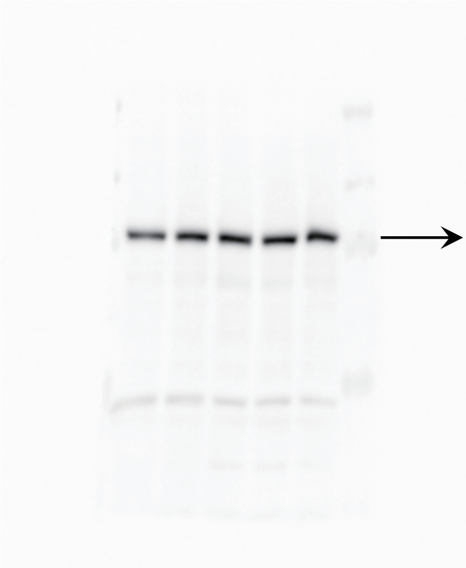

→ S552-β-catenin

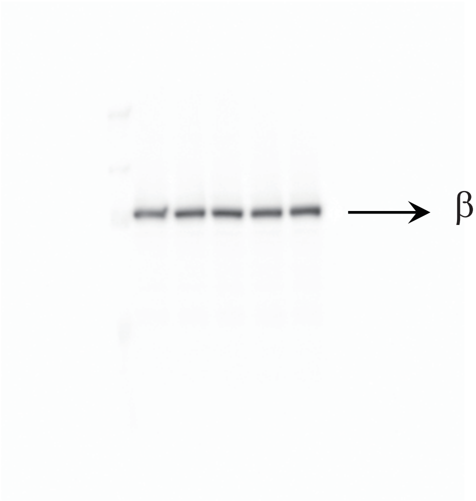

→ β-catenin

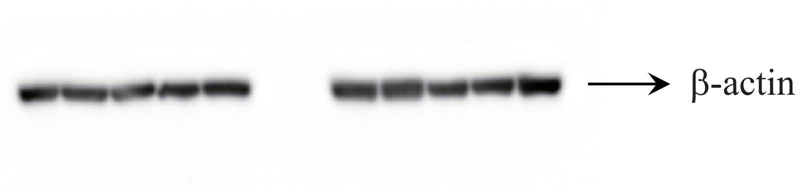

→ β-actin
